# An Overview of Lsr2 Repressor Effect in *Streptomyces* spp. Secondary Metabolism

**DOI:** 10.3390/microorganisms12112317

**Published:** 2024-11-14

**Authors:** Lorena Cuervo, Mónica G. Malmierca, Carlos Olano

**Affiliations:** 1 Functional Biology Department, University of Oviedo, 33006 Oviedo, Spain; 2 University Institute of Oncology of Asturias (I.U.O.P.A), University of Oviedo, 33006 Oviedo, Spain; 3 Health Research Institute of Asturias (ISPA), 33006 Oviedo, Spain

**Keywords:** *Streptomyces* spp., Lsr2, secondary metabolites

## Abstract

The genus *Streptomyces* is one of the largest producers of secondary metabolites with bioactive properties of interest. However, many of the genes involved in synthesizing these compounds are silenced under laboratory conditions. One of the strategies used to activate these metabolic pathways is the elimination of repressor genes, which prevent the transcription of other genes. In this work, the *lsr2* gene has been selected for study since it is a repressor with a preference for binding to AT-rich regions, which makes it exert its effect especially on those horizontally transferred gene sequences that have a very different GC content to the core *Streptomyces* spp. genome. Therefore, the effects of the deletion of this gene are observed, and, in addition, a mapping of the potential binding sites of Lsr2 in *Streptomyces* spp. genomes is proposed. As a result of this gene knockout, the production of various secondary metabolites is overproduced and/or activated, which suggests that the study of this regulator can be interesting in the field of natural product discovery.

## 1. Introduction

Bacteria use a multitude of mechanisms to improve their survival in the changing surroundings in which they are found: spore formation, changes in the cell membrane, biofilm formation, etc. [1,2,3]. One of these strategies is the production of compounds that provide selective advantages in the environment, such as antibiotics or siderophores, which improve their adaptability to stressful environments. Furthermore, despite the fact that many of the biosynthetic genes involved in their production are silenced in laboratory conditions, leaving their metabolites unknown [4,5], these compounds have enormous relevance because they have numerous applications in the food and pharmaceutical industries, bioremediation, etc. [6]. Another strategy that enhances the fitness of the organisms is horizontal gene transfer (HGT), which promotes genetic diversity. This mechanism is the basis of bacterial evolution along with mutation, genetic drift, selection, and dispersal [7,8,9,10]. Either small fragments of DNA or several groups of genes can be transferred [10]. Nevertheless, there is also the possibility that horizontal gene transfer will diminish the competitive advantages interfering with other regulatory pathways. This reveals how cellular genetics is not only influenced by the inheritance of genetic material vertically but is also related to the horizontal transmission of DNA from organisms that may be phylogenetically distant, resulting in mosaic genomes [10,11,12]. In this regard, among all the effects that the acquisition of xenogenetic genes can trigger, the gaining of those involved in secondary metabolism is highlighted.

Great interest is placed in the study of secondary metabolites, particularly those produced by exogenous genes as well as those produced by silenced genetic clusters: the development of resistance to current drugs as well as the emergence of new diseases has ended up claiming a need for research into new natural products with bioactive properties such as antibiotics, antifungals, cytotoxic agents, etc. The study of these biosynthetic gene clusters (BGC) could open the door to producing unknown bioactive compounds with potential interests that could serve as therapeutic alternatives [4].

There are various strategies to attempt to activate silenced metabolic pathways, including the overexpression of global regulators or group-specific genes, heterologous expression, or deletion of repressors [13,14]. In this context, we highlight the role of some bacterial nucleoid-associated proteins (NAPs) in gene regulation, especially the horizontally acquired ones. NAPs are small polypeptides involved in the organization of nucleoids and the regulation of genetic expression. Through different mechanisms, they interact with the DNA structure, modifying the shape of the DNA by bending, wrapping, or bridging it in the nucleoid and consequently influencing transcription positively or negatively [15,16]. In this way, a balance is regulated between the compaction of DNA and its accessibility to protein complexes involved in cellular processes [8]. Lsr2 proteins belong to this group of NAPs that cause transcriptional repression and bind preferentially to AT-rich regions of the genome through interactions with the minor groove of DNA [17]. Lsr2-type proteins are found in actinomycetes in contrast to the other three xenogeneic classes of proteins, which can recognize foreign DNA elements and with which it shares similarities in the oligomerization and DNA-binding domains: H-NS-like proteins in proteobacteria, MvaT/U-like in Pseudomonodales, and Rok in diverse bacilli [9]. Due to the elevated level of GC content that characterizes Actinobacteria, Lsr2 proteins tend to bind to horizontally acquired DNA motifs since they present distinct content of nucleobases notably in comparison to the rest of the genome. The repressive effect of this protein is not solely restricted to horizontally transferred genes, so it may also affect the expression of other BGC present in the corresponding genome. This silencing effect is usually caused by the occlusion or the trapping of RNA polymerase, due to the interference with the transcription elongation complex, or improving termination [9,18,19].

This group of proteins is small and conserved in actinomycetes, and the changes generated in the genome can be substantially significant regarding metabolic production while in other cases they are not so considerable [17]. Not only can they exert effects on genes that control secondary metabolism, but also on those related to the cell wall or the acquisition of antimicrobial resistance [17]. It has been described that the deletion of *lsr2* drives the positive regulation of some repressed groups of genes, therefore, its elimination would be interesting to discover new bioactive compounds that are silenced in the genome of the strains.

Given the significant interest that actinomycetes present as a group with biosynthetic potential [20,21,22], the present research is focused on the impact of this repressor on the well-known genus *Streptomyces*, since it is the largest producer of secondary metabolites with properties of interest. Alongside, an attempt has been made to map the potential binding sites of Lsr2 in the BGC responsible for the production of compounds that have been affected as a consequence of the elimination of the repressor gene, either by an increase in the production of these compounds or by the activation of silenced metabolic pathways. In addition, we used the in-house CS streptomycete collection (named after Carlos Sialer) for this study. These strains were isolated from the tegument of ants from the *Attini* tribe. The interest in using these strains relies on the fact that they were isolated from an unexplored environment, the ants′ nests, which represents an attractive field in the search for novel bioactive compounds.

## 2. Materials and Methods

### 2.1. Strains and Culture Conditions

In this work, five strains of *Streptomyces* spp. of the CS collection isolated from leaf-cutting ants of the *Attini* tribe of Perú [23,24] were employed. The strains were cultured at 30 ºC for 7 days in Medium A [25], and spores were scratched from the surface and stored at −80 °C in 20% glycerol. *Escherichia coli* ET12567 carrying pUZ8002 was grown in 2xTY (tryptone yeast) with the appropriate antibiotics and subsequently used for intergenic conjugation as described in [26], employing SFM (Soy Flour Mannitol) with 0.1 mM MgCl_2_ [26]. For the metabolite production, precultures were grown in Tryptic-Soy Broth and after 24 h, cultured in R5A [27], SFM [28], YMA [29], or SM10 [23] media (all of them in their liquid version).

### 2.2. Design and Plasmid Construction

To evaluate the effect of *lsr2* in the different strains, the knockout of this gene was designed by replacement. Homologous fragments were amplified in each strain (primers used in Supplementary Materials, Table S1) and cloned into pUO9090 [30] by restriction-ligation for the construction. Thereafter, these fragments were digested with BcuI and cloned together in bloc into pHZ1358 [31] to perform recombination. pUO9090 encodes the aminoglycoside acetyl-transferase gene that confers resistance to apramycin and tobramycin, while pHZ1358 has the ampicillin and thiostrepton resistance genes. The final construction of pHZ1358 generated for each strain contains the two fragments adjacent to the corresponding *lsr2* along with the apramycin resistance gene. The different plasmids were transferred by electroporation to *E. coli* ET12567 carrying pUZ8002 cells and subsequently transferred by conjugation into *Streptomyces* strains. In the latter, efficient recombination resulted in the deletion of *lsr2* and the introduction of the apramycin resistance gene into the corresponding location. The mutants obtained were checked by PCR (primers used in Supplementary Materials, Table S1) and sequencing. A schema of the cloning steps is shown in Figure S1 in Supplementary Materials (SnapGene^®^ 7.0 software was used to create the images).

### 2.3. Metabolite Production and Chromatographic Analysis

As a control, during the evaluation of the production profiles of the mutant strains, the different strains containing the pSETxk vector were used to rule out the changes that can be caused by the simple acquisition of apramycin resistance, an approach that has been used previously [32]. For metabolite production, strains were cultured 24 h on Tryptic-Soy Broth in baffled flasks at 30 °C and 250 r.p.m. After that, from these pre-cultures, non-baffled flasks were inoculated containing 50 mL of different media until reaching an O. D. = 0.2. Samples of the same cultures were taken after 3, 5, and 7 days of growth and extracted with three different organic solvents: ethyl acetate, acidic ethyl acetate (1% formic acid), and butanol. After 1–2 h of mixing, the solvent phase was separated by centrifugation and dried under vacuum (Labcono CentriVap Benchtop Vacuum Concentrator, Kansas City, MO, USA). Also, samples of the cultures were taken and dried at 60 °C to calculate the dry mass weight. Finally, the dried samples from the solvent extraction were resuspended on methanol in proportional volume to the mass of the dry weight of the mycelia. Ten microliters were injected into the UPLC for chromatographic analysis and run in an Acquity UPLC I-Class (Waters, Milford, MA, USA) using a BEH C18 column (1.7 μm particle size, 2.1 mm × 100 mm) and acetonitrile and water containing 0.1% of trifluoroacetic acid as mobile phase. A gradient was used from 10 to 99% of acetonitrile in 10 min and a flow rate of 0.5 mL/min. For HPLC/MS analysis, a Waters ZQ4000 system (Milford, MA, USA) was used connected to an HPLC 2695/2795 (An Alliance chromatographic system coupled to a SunFire C18 column −3.5 μm particle size, 2.1 mm × 150 mm- and a 996 PDA detector scanning wavelengths between 200 and 600 nm) (Milford, MA, USA). Acetonitrile and MQ water + formic acid 0.1% (ThermoFisher, Waltham, MA, USA) were used as the mobile phase and elution was performed with an isocratic hold with acetonitrile (10%) for 1 min followed by a linear gradient of acetonitrile (10–100%) over 7 min (0.5 mL/min). The Empower 3.0 (Milford, MA, USA) program was used to compare and analyze the chromatograms obtained from each sample. Each test was performed in triplicate.

### 2.4. Dereplication Analysis

HRMS-based dereplication was performed at Medina Foundation using their in-house library and the Dictionary of Natural Products version 26:2 to identify known compounds. LC–MS was performed on Agilent 1200 Rapid Resolution HPLC and analysis was performed on a maXis Bruker qTOF mass spectrometer (Santa Clara, CA, USA). The volume injected was 2 µL and a Zorbax SB-C8 column (Santa Clara, CA, USA, 2.1 × 30 mm, 3.5 µm particle size) was used for the separation. The mobile phase consisted of solvent A, 90:10 milliQ water–acetonitrile, and solvent B, milliQ water–acetonitrile, both with 13 mM ammonium formate and 0.01 TFA. Samples were eluted with a 0.3 mL/min flow rate, and the gradient used was 90% to 0% solvent A/10% to 100% solvent B in 6 min, 0% solvent A/100% solvent B in 2 min, 0% to 90% solvent A/10% to 100% solvent B in 0.1 min, and 90% solvent A/10% solvent B for 9.1 min. The maXisqTOF mass spectrometer was operated in ESI-positive mode. Source conditions were 4 kV capillary voltage, end plate offset = 500 V, dry gas (N_2_) flow = 11 L/min; dry temperature = 200 °C, and nebulizer (N_2_) pressure at 2.8 bars. The retention time, together with the exact mass and the derived molecular formula, was used as the criteria to search in databases. Data concerning the characterization of compounds by LC–MS are available in Supplementary Materials: LC–MS dereplication.

### 2.5. Bioinformatic Tools

NCBI public access database was used for the search of *lsr2* sequence from *Streptomyces venezuelae* ATCC 10712 (accession number NC_018750), and subsequently, the BLASTn tool was performed to compare against genomes of the CS collection to delimitate the regions with the greatest homology. Accession numbers for the genomes are NZ_KZ819154 for CS014, NZ_KZ195572 for CS057, NZ_KZ819167 for CS065a, NZ_KZ819178 for CS131, and NZ_KZ195583 for CS227.

AntiSMASH 7.0 software [33] was used to identify the potential secondary metabolites gene clusters present in CS collection genomes, considering that a prediction is reliable when the percentage of identity is greater than 85%.

FIMO (Find Individual Motif Occurrences) software [34] (MEME Suite 5.5.7) was used to determine the location of potential binding sites present in the sequence of the genome of the *Streptomyces* spp. strains. Based on the work presented in [35] about potential binding sequences to Lsr2, 18 motifs (Table 1) from the 136 motifs that present 100% A-T content were selected for the analysis. We consider that the motif analysis presented in [35] is extrapolable to *Streptomyces* due to the fact that the nucleoid-associated protein Lsr2 is conserved throughout the Actinobacteria phylum [9]. The motifs were selected among the highest and most frequent E-score and Z-score values, thus discriminating those isolated values further apart from the group media: E-score values between 0.45 and 0.46 and Z-score values were selected between 6.45 and 7.63. This analysis was done for the five strains of *Streptomyces* spp. Tested, and the motifs detected with a *p*-value less than 0.0001 were observed, which is the default calculation of the software.

## 3. Results

Once the knockouts of the *Streptomyces* strains CS014, CS057, CS065a, CS131, and CS227 were verified (by PCR and sequencing), they were grown in different culture media with variable sources of carbon and nitrogen. Samples were taken from different culture broths and extracted with three different solvents (butanol, ethyl acetate, and ethyl acetate containing 1% formic acid). The samples were dried and then resuspended in methanol according to their weight. Notably, the weight of the mutants and control strains was found to be similar, indicating that the gene loss did not significantly impact bacterial growth. The profiles of metabolite synthesis were compared to those of the strain harboring the empty vector pSETxk. A qualitative comparison of the chromatograms was made.

### 3.1. Streptomyces sp. CS014

**Figure 1:**
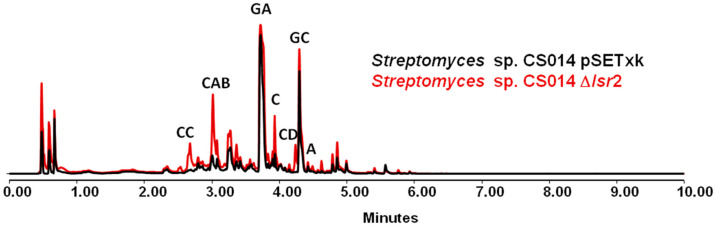
UPLC analysis of samples of *Streptomyces* sp. CS014 strains cultured in SM10 media and extracted with ethyl acetate containing 1% formic acid at 5 days of growth. It can be seen the activation of the synthesis of CC (collismycin C) and CD (collismycin D) and the overproduction of CAB (collismycin A-B), GA (granaticin A), C (coproporphyrin), GC (granaticin C), and A (aloesaponarin II).

The culture of strain CS014 Δ*lsr2* in the different media reveals different metabolic changes, although almost all of them tend to be common, varying the amount of compound produced as well as different profiles throughout the different timings sampled. On the one hand, one group of compounds affected is the collismycins [32,36]. Collismycin C is activated in all media in the mutant, while this compound is not produced by the control strain under the conditions tested. The forms identified by dereplication (comparing retention times, exact mass, and derived molecular formulas with databases) as collismycins A-B show a very large increase in production (in some cases, by more than 10-fold) (Figure 1). In some cultures, the activation of collismycin A was also observed. The production of granaticins is also affected, showing an overproduction of granaticins A and C [37]. Different coproporphyrins [38] are also overproduced in large quantities in the mutant strain. In specific cases, the overproduction of cyclo (Tyr-pro) [39] in R5A medium (ethyl acetate and ethyl acetate containing formic extracts) or aloesaponarin II in SM10 (ethyl acetate extract on day 7) were also identified [40].

From the 18 motifs analyzed using the FIMO software (MEME Suite 5.5.7) [34], nine of these sequences were detected with 17 occurrences in the genome of *Streptomyces* sp. strain CS014. For motif 14 (AAATATTT), four occurrences are detected in two locations owing to the fact that the program detects this sequence in both strands since the sequence complementary to this motif is the same (AAATATTT). Therefore, only two occurrences were considered for this motif. In Table S2 of Supplementary Materials, a summary of the locations of the remaining 15 motif occurrences is detailed. Motifs 6 (AAATAAAT, only located in 1203755-1203762 nt) and 7 (AATTAAAT) are located in an intergenic area within the cluster of collismycins biosynthesis based on the predictions offered by AntiSMASH 7.0. The rest of the motifs are found between clusters or in scaffolds with no presence of BGCs.

### 3.2. Streptomyces sp. CS057

**Figure 2:**
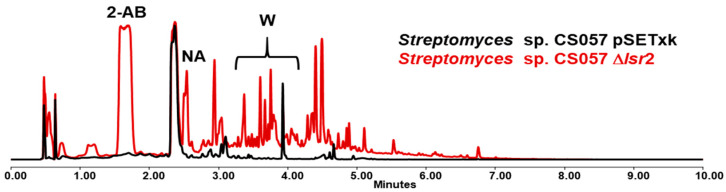
UPLC analysis of samples of *Streptomyces* sp. CS057 strains cultured in R5A media and extracted with ethyl acetate at 3 days of growth. 2-AB indicates the activation of the production of 2-aminobenzoic acid, NA the activation of nonanoic acid biosynthesis, and W the new synthesis of warkmycins. The activation of other compounds that could not be identified was observed.

**Figure 3:**
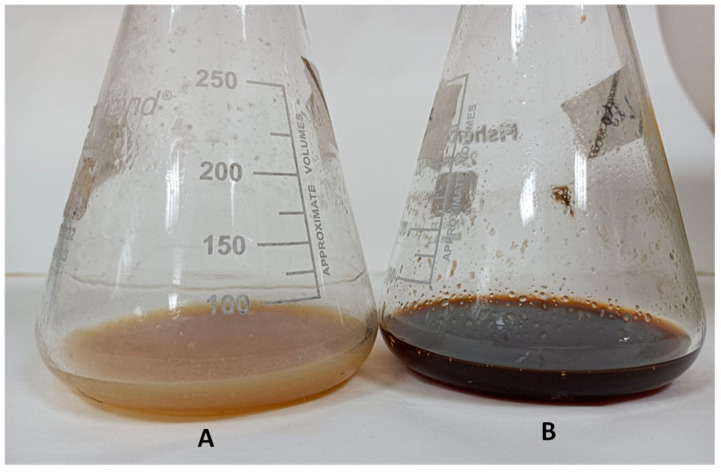
Activation of warkmycins due to deletion of the *lsr2* gene in *Streptomyces* CS057. (**A**) *Streptomyces* CS057 pSETxk; (**B**) *Streptomyces* CS057 Δ*lsr2*.

The cultivation of CS057 Δ*lsr2* shows quite a different profile compared to the control strain that contains the empty vector pSETxk. The comparison of the results collected with other databases allowed the identification of various metabolites. The activation of warkmycin biosynthesis [41] in all media tested can be appreciated (Figure 2). Due to the dark brown color of warkmycins, this activation can be appreciated visually (Figure 3). Additionally, 2-aminobenzoic acid [42] and nonanoic acid production [43] were activated in the R5A medium. The activation of the production of other compounds that could not be identified by dereplication was also detected. However, although the nature and activity of these unknown compounds are of great interest, this is outside the scope of the present work and might be pursued in the future, since we only intend to evaluate the effect of the *lsr2* gene on the metabolism of *Streptomyces* spp.

From the 18 motifs analyzed, nine of these sequences were detected with 22 occurrences in the genome of *Streptomyces* strain CS057. For motif 14 (AAATATTT), six occurrences are detected in three different locations. Therefore, as in the previous sample, only three occurrences were considered. In Table S3 of Supplementary Materials, a summary of the locations of the remaining 19 motifs occurrences is detailed. Motif 9 (AATAAATT) is located in the gene ctg1_6972 of cluster 1.35, which encodes for a methyltransferase. Motif 8 (AAATAATT) is located in two intergenic regions, within the clusters 1.9 (position 897433-897440 nt) and 1.32 (position 7963726-7963733) involved in SGR PTMs and naringenin biosynthesis based on the predictions of AntiSMASH. The rest of the motif occurrences detected are between clusters or in scaffolds with no BGCs.

### 3.3. Streptomyces sp. CS065a

**Figure 4:**
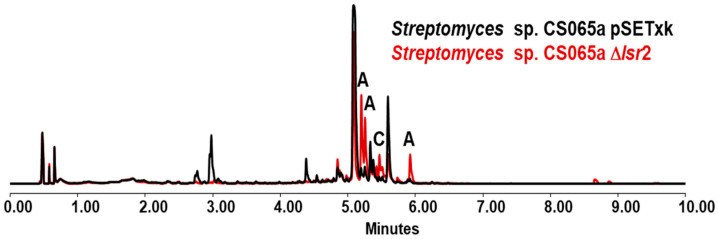
UPLC analysis of samples of *Streptomyces* sp. CS065a strains cultured in R5A media and extracted with ethyl acetate at 5 days of growth. A indicates the overproduction of alteramides and C the overproduction of chromomycins.

The cultivation of mutant CS065a Δ*lsr2* reveals an increase in the production of alteramides in all media tested (Figure 4) [29,44], with differences depending on the extracts and sampling times in each culture medium. In addition, biosynthesis of chromomycin-type compounds (chromomycin A_3_, A_p_, A_2_) [45] is also positively affected upon deletion of *lsr2*: in all the media tested, except SFM media, an overproduction of these metabolites is shown (Figure 4). The overproduction of coproporphyrins as well as other metabolites not identified by the analytical methodology followed is also observed in this strain. All these microbial metabolites have been identified thanks to dereplication analysis.

From the 18 motifs analyzed, eight of these sequences were detected with 16 occurrences in the genome of *Streptomyces* strain CS065a. For motif 14 (AAATATTT), four occurrences are detected, and as in the previous strains, only two were considered. Therefore, in Table S4 of Supplementary Materials, a summary of the locations of the remaining 14 motifs occurrences is detailed. In this strain, all the motif occurrences detected are between clusters or in scaffolds with no BGCs.

### 3.4. Streptomyces sp. CS131

**Figure 5:**
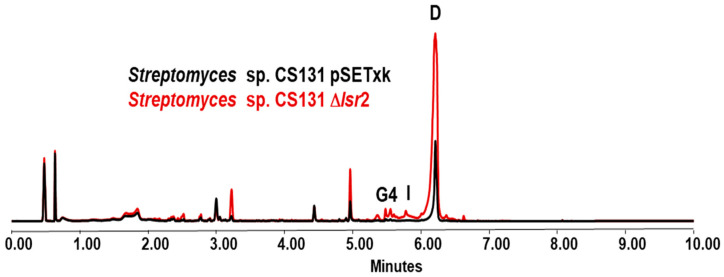
UPLC analysis of samples of *Streptomyces* sp. CS131 strains cultured in R5A media and extracted with ethyl acetate at 3 days of growth. D indicates the overproduction of actinomycin D; G4 and I indicate the activation of the production of actinomycin and G4 and I, respectively. The production of actinomycin D in these conditions is a 4.8% increase by the mutant in comparison to the control strain.

The analysis of CS131 Δ*lsr2* extracts compared to that of the control strain shows an increase in the production of actinomycin D [46] at different levels in all media tested, except for the YMA medium in which no differences were observed. The most pronounced increase in production was observed in the SM10 medium, where production reaches its maximum on day 5 of culture, and the extraction of metabolites with the solvent butanol shows an increase of 189% in comparison to the control strain. Since actinomycin is a compound already described and widely studied, the identification of its production has been clear. However, both in R5A and SM10, the increase in production of other compounds not identified by the dereplication methodology performed is also observed. Likewise, activation of the production of actinomycin G4 and actinomycin I was detected in the R5A (Figure 5) and SM10 media.

From the 18 motifs analyzed, 12 of these sequences were detected with 16 occurrences in the genome of *Streptomyces* strain CS131. For motif 14 (AAATATTT), two occurrences are detected, and as in the previous strains, only one was considered. Therefore, in Table S5 of Supplementary Materials, a summary of the locations of the remaining 15 motifs occurrences is detailed. Motif 14 is located in cluster 1.1, in the intergenic region between genes ctg1_36 and ctg1_37. The remainder of the motif occurrences detected are located between clusters or in scaffolds with no BGCs.

### 3.5. Streptomyces sp. CS227

**Figure 6:**
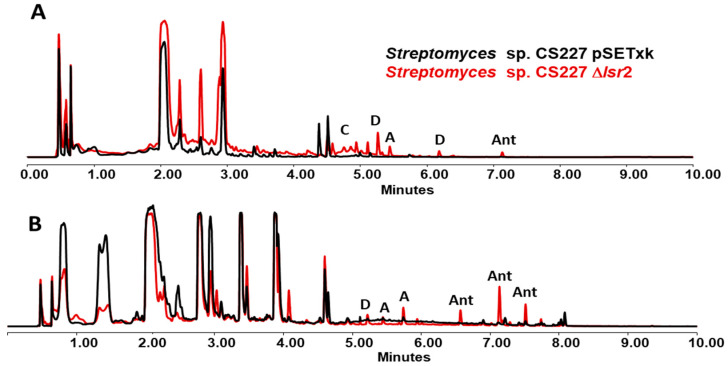
UPLC analysis of samples of *Streptomyces* sp. CS227 strains cultured in SFM media and extracted with ethyl acetate containing 1% formic acid. (**A**) Samples of 3-day growth cultures. (**B**) Samples of 5-day growth cultures. Depending on the day of cultivation, different metabolic production is observed. Activation of dihydromaltophilin (D), overproduction of candicidins (C), antimycins (Ant), and alteramides (A) are shown.

The metabolic production profile of the mutant strain CS227 Δ*lsr2* is quite different from that of the control strain (Figure 6). The activation of antimycin production [47] is detected in R5A and YMA media, although in a very low quantities. In SFM, this compound is overproduced. In the three media previously mentioned, an increase in the production of candicidins [48] and alteramides [44], and the activation of dihydromaltophilin [49] production is also detected. A database comparison of the results performed has allowed the identification of these metabolites.

From the 18 motifs analyzed, six of these sequences were detected with nine occurrences in the genome of *Streptomyces* strain CS227. For motif 14 (AAATATTT), two occurrences are detected in the same location, so only one was considered. Therefore, in Table S6 of Supplementary Materials, a summary of the locations of the remaining eight motifs occurrences is detailed. Motif 13 is located in cluster 1.13, the intergenic region between genes ctg1_orf04382 and ctg1_orf04384. The remainder of the motif occurrences detected are located between clusters or in scaffolds with no BGCs.

## 4. Discussion

The activation of silenced metabolic pathways in microorganisms is one of the hot spots in natural product research, with the goal of isolating and characterizing new compounds as well as producing novel derivatives. One of the most used approaches focuses on the elimination of repressors since, in many cases, they involve the release of the pathways from their blocked state, which in turn leads to the production of compounds [50]. Lsr2 proteins act as xenogeneic silencers repressing the expression of genes acquired by horizontal transfer, actively discriminating between own sequences and those acquired due to variations in GC content. Thus, this regulator represses the expression of genes that could be involved in specialized metabolism and therefore the production of interesting bioactive compounds [51,52]. Understanding the regulatory processes of this protein is particularly important because it may act as a trigger for the synthesis of metabolites, which the pharmaceutical sector may employ to develop therapeutic alternatives to existing treatments [53,54]. Various factors can influence the observation of results at the experimental level. Primarily, the optimal culture conditions for the expression of genes that have been previously suppressed should be determined. Conversely, it is feasible that in vivo the binding of particular transcription factors counteracts the repressive effect of Lsr2, which might mean that in *lsr2* mutants produced as a result of this counter-silencing, metabolic alterations might go undetected in experiments [54].

Since Lsr2 is a conserved regulator throughout the Actinobacteria phylum, its effect on gene expression has also been described in other microorganisms. In this work, we have focused on analyzing a collection of streptomycete strains isolated from a very particular environment. However, most studies regarding this protein have been performed on *Mycobacterium* spp. [55], describing not only its involvement in secondary metabolism and cell cycle [56] but also as a mediator in response to stress and determinants in virulence. The latter is potentially interesting considering the incidence of tuberculosis in the world population and consequently the interest in developing anti-tuberculosis drugs [57,58]. This also gives us an idea of the multiple implications that Lsr2 may have in streptomycetes not related to secondary metabolism. Homologated genes have been reported in other phylum-sharing organisms such as *Nocardia, Rhodococcus,* or *Micromonospora*, although no studies have been described in reference to this repressor [59,60]. More studies are needed to clarify the regulatory framework under Lsr2, although it certainly presents itself as a hot spot of great and promising interest.

As a whole, the complexity of the mechanisms of action of Lsr2 remains unclear, making it an attractive field of study, since it can also interfere with different cellular aspects such as metabolism, stress responses, or life cycle. Summers and colleagues [61] suggest a mechanism for Lsr2 oligomerization through protease activation leading to chromosome compaction and protection and subsequent gene repression. Other paralogues have also shown some similarities in mechanisms such as nucleoid-organizer and protein regulator. For example, H-NS in gram-negatives has shown interactions with itself, generating homodimers and oligomers that regulate expression. The state of repression arises when DNA is bound with high affinity, generating a higher-order nucleoprotein complex that traps the RNA polymerase or occludes its binding [53]. On the other hand, Rok proteins have a slightly different mechanism than classical xenogeneic silencers, as they bind to the small groove of DNA through a winged helix motif that generates oligomers [61].

In this work, we generate mutants deficient in the *lsr2* gene in five *Streptomyces* spp. strains from the CS collection, and cultures were performed in different culture media with different carbon and nitrogen sources [54]. These strains, isolated from a little-explored environment such as the integument of leaf-cutter ants, arouse great study interest given the possible bioactive secondary metabolites they can produce, including novel and unknown structures [62]. Furthermore, previous bioinformatics studies performed on these strains revealed the enormous biosynthetic potential of this collection [63]. In this way, it is shown how Lsr2 exerts a repressive effect on certain metabolic pathways since its elimination activates or increases their expression, which could be an interesting tool to explore in drug discovery programs [64].

On the other hand, we have tried to predict bioinformatically the effects of the removal of this gene on the metabolic outcomes of the mutant strains. A DNA motif is a short and conserved sequence that may correspond to a protein binding site, being a key unit of molecular evolution [34]. The FIMO software allows the scanning of both DNA and protein sequences to identify binding sites or protein motifs, performing as a novel and easily accessible tool. The results obtained from this analysis offer a list of motifs found in a sequence as well as their location with a likelihood ratio score, using a *p*-value threshold of 0.0001 [34].

In this work, 18 sequences from those exposed by Wiechert and colleagues [9] were selected as potential binding sites for Lsr2. These 18 selected motifs presented 100% AT content, so they are considered the most probable binding points for the repressor protein. Based on this, the position of these sequences in the genome of each of the strains tested has been determined. Most of these motifs occur in areas between BGCs or in scaffolds that do not present BGCs at all. However, other sequences were found located in certain biosynthetic regions. The case of the *Streptomyces* sp. CS014 strain is a great predictive example. On the one hand, two of these practically overlapping motifs are located between two collismycin biosynthesis genes. Collismycins are one of the secondary metabolites that are significantly affected by the deletion of *lsr2,* since the activation of forms A and D is predominantly observed, as well as an enhancement in the production of form AB. Although no binding points have been detected on the genes themselves but between them (intergenic areas), this area may be affected by the effects of a promoter as it is released of the blocking state as a result of the deletion of *lsr2*. The observed effect is similar to what happened with the heterologous expression of *pimM* in *Streptomyces clavurigerus*, which gives rise to the overproduction of up to 7-fold more cephamycin C and about 10-fold more clavulanic acid since binding points of this activator were detected in the intergenic region upstream of *ccaR*, a global regulator with influence on the BGC of those secondary metabolites. Thus, in that case, the binding in the intergenic zone under the influence of a promoter triggers changes in secondary metabolism [65].

In a similar way, collismycin BGC likely presents low expression conditions since the deletion of the repressor gene increases the production of compounds and activates the production of new ones, which otherwise might simply be produced in wild-type strains at minimal undetectable concentrations under the analytic approaches performed.

In the case of the other BGCs that present binding points, no experimental changes were observed to support the findings, probably because the conditions tested are not optimal for the production of those compounds, because they cannot be detected by the analysis performed, or simply because the presence of a binding point does not necessarily have to trigger a response or to affect secondary metabolism. In this sense, neither naringenin nor SGR PTMs were detected. As mentioned, the conditions for the detection of those compounds may not have been optimal. For example, the production in other media like SA (for naringenin) [66] and MYG media (for SGR PTMs) [67] has been described in other *Streptomyces*. This may imply that genetic manipulation techniques may not be sufficient in the absence of minimum nutritional requirements.

On the other hand, we do not know if these BGCs are subjected to genetic control by Lsr2, so the deletion may not imply metabolic changes that affect these productions, since the metabolic network and the regulatory cascades that affect a specific BGC are complex and many remain unknown [20,68]. Further work is necessary to elucidate this point. For example, a transcriptomic study could quantify the effect of the *lsr2* deletion on putative biosynthetic genes [69]. On the other hand, the low homology offered by AntiSMASH for cluster 1.35 in *Streptomyces* CS057 and 1.1 in *Streptomyces* CS131, as well as the lack of homology for cluster 1.13 for *Streptomyces* CS227, does not allow us to draw conclusions, since they could be affecting the synthesis of any metabolite already described or not. It has indeed been observed that changes in the production of compounds in which we have not detected binding sequences at the corresponding BGCs. These can be easily explained since, on the one hand, we have only selected 18 potential union points in this work, those most promising. Another likely explanation is that the effect of the repressor is exerted on regulatory elements encoded by genes located outside the particular BGC. Thus, the overproduction/activation of compounds not identified by dereplication has also been detected.

As a whole, these results show how the knockout of the Lsr2 repressor coding gene can be a non-targeted strategy to explore the discovery of new natural products [55,56,57,58,59,60]. In addition, it is interesting to be able to obtain more information about how this response can affect multiple pathways of secondary and primary metabolism. Although this work only evaluates the metabolic production profile, effects on growth as a consequence of the deletion of *lsr2* have already been described [52], which advocates the complex metabolic network in which this repressor participates. Finally, FIMO software, which has been used in various research studies in *Streptomyces* [70,71,72], appears to be a useful tool in the study of metabolic pathways that perfectly complements experimental tests and can help decipher the complex role that a protein can have in the metabolic regulation of organisms.

## 5. Conclusions

The knockout of the repressor gene *lsr2* in various *Streptomyces* strains results in an increase in the production of secondary metabolites and leads to the activation of silenced metabolic pathways. Furthermore, FIMO software is an interesting tool to map the binding sites of the repressor and consequently could be used to predict the effects of its elimination. Without a doubt, this study highlights the complex metabolic framework underlying *lsr2* and presents its deletion as an interesting point to consider in drug discovery programs.

## Figures and Tables

**Table 1:** Motifs selected for the analysis.

Motif 1: ATTTAAAT	Motif 7: AATTAAAT	Motif 13: TATATTAA
Motif 2: AATTTAAT	Motif 8: AAATAATT	Motif 14: AAATATTT
Motif 3: TAATTTAA	Motif 9: AATAAATT	Motif 15: ATATAATA
Motif 4: AATTTAAA	Motif 10: ATTATAAT	Motif 16: TAAAATAA
Motif 5: ATATTAAT	Motif 11: AATAAATA	Motif 17: TTAATTAA
Motif 6: AAATAAAT	Motif 12: AATTATAA	Motif 18: AAATTATA

## Data Availability

The original contributions presented in the study are included in the article/Supplementary Materials, further inquiries can be directed to the corresponding author.

## Supplementary Materials

The following supporting information can be downloaded at: https://www.mdpi.com/article/10.3390/microorganisms12112317/s1, Table S1: primers used for plasmid construction and mutants check; Figure S1: outcome of the construction of the different plasmids (Figures made by SnapGene^®^ 7.0); Table S2: motifs located in *Streptomyces* sp. CS014; Table S3: motifs located in *Streptomyces* sp. CS057; Table S4: motifs located in *Streptomyces* sp. CS065a; Table S5: motifs located in *Streptomyces* sp. CS131; Table S6: motifs located in *Streptomyces* sp. CS227.
