# Supplementary material for: An Overview of Lsr2 Repressor Effect in Streptomyces spp. Secondary Metabolism
Source: Microorganisms. 2024 Nov 14;12(11):2317. doi: 10.3390/microorganisms12112317 (PMC11596768; doi:10.3390/microorganisms12112317)

Supplementary Materials

Table S1: primers used for plasmids construction and mutants check.

Strain	Primer	Amplification
Streptomyces sp. CS014	3′-TATGAATTCGTTTTCGGCTCGCTACTCTG-5′	Amplification
	3′-TATAAAGCTTCGACAAGAAGGACCTGAACC-5′	
	3′-TATCATATGTCCGTGAGGCTTACGAGAAG-5′	Amplification
	3′-TATTCTAGAGGGATTCTTACTGCCGAACA-5′	
	3′-CTGGAGCGCTGTCAGGTG-5′	Mutant check
	3′-CGGTGAACCTCTCGAACATC-5′	
Streptomyces sp. CS057	3′-TATGAATTCTGGACGGCATGGACTATGTA-5′	Amplification
	3′-TATAAGCTTCGAGGTCATCGACAAGAAGG-5′	
	3′-TATGGATCCAGGCTTACGAGAAGGCCAAC-5′	Amplification
	3′-TATTCTAGACGATGAACAGGATGATGTCG-5′	
	3′-CGGCTACCTGGAACTGCTC-5′	Mutant check
	3′-TTGAGGATCTCGATGGTGTG-5′	
Streptomyces sp. CS065a	3′- TATGAATTCCTCTGGACGGCATGGACTAC-5′	Amplification
	3′- TATAAGCTTCGACAAGAAGGACCTGAACC-5′	
	3′- TATCATATGAGGCTTACGAGAAGGCCAAC-5′	Amplification
	3′- TATTCTAGACGATGAACAGGATGATGTCG-5′	
	3′- CTCCGTCACCGGCTATCTG-5′	Mutant check
	3′- GCGATCTTCTCGTCGAACTC-5′	
Streptomyces sp. CS131	3′- TATGAATTCCTCTGGACGGCATGGACTAC-5′	Amplification
	3′- TATAAGCTTCGACAAGAAGGACCTGAACC-5′	
	3′- TATCATATGTCCGTGAGGCTTACGAGAAG-5′	Amplification
	3′- TATTCTAGATGCTTGTCCTTGAGGGTCTC-5′	
	3′- GGGTGTAGAGGTGCTTGTCC-5′	Mutant check
	3′- CACCGGTTATCTGGAGCTG -5′	
Streptomyces sp. CS227	3′-TATGAATTCAGGGGTGATCCCTTTTCGT-5′	Amplification
	3′-TATAAGCTTCGACAAGAAGGACCTGAACC-5′	
	3′-TATGGATCCCCGAGTACGAGAAGGCCAAC-5′	Amplification
	3′- TATTCTAGATCCAGGGTGTAGAGGTGCTT-5′	
	3′-CTCATCGTGACCATCGTGAA-5′	Mutant check
	3′-AGCTCGTCGATGAACAGGAC-5′	

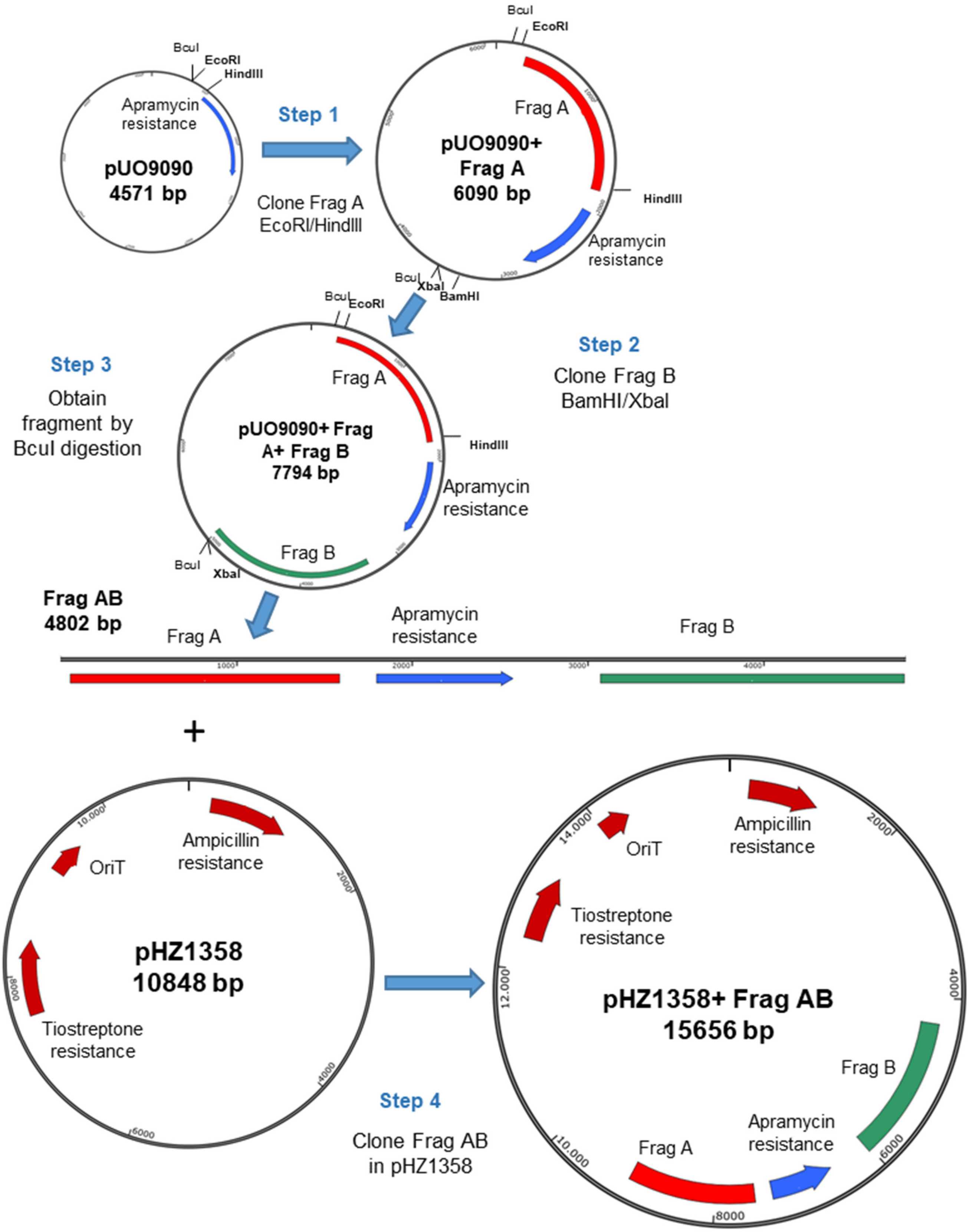

Figure S1: outcome of the construction of the different plasmids (Figures made by SnapGene®).

Table S2: motifs located in *Streptomyces* sp. CS014.

Motif	Strand	Position	Scaffold	Placed in	Within a BGC*
16	+	4671024-4671031	1	Between clusters 1.16 (T2PKS-granaticin) and 1.17 (NI-siderophore-desferrioxamin B)	-
16	+	4843502-4843509	1	Between clusters 1.16 (T2PKS-granaticin) and 1.17 (NI-siderophore-desferrioxamin B)	-
14	+/-	194-201	4	Before cluster 4.1 (NRPS/T2PKS)	-
14	+/-	4546751-4546758	1	Between clusters 1.16 (T2PKS-granaticin) and 1.17 (NI-siderophore-desferrioxamin B)	-
11	+	7036233-7036240	1	Between clusters 1.20 (terpene) and 1.21 (terpene-isorenieratene)	-
9	-	1203754-1203761	1	In cluster 1.7 between genes *ctg1_1004* and *ctg1_1005*	Collismycin A (77%)
8	+	1878903-1878910	1	Between clusters 1.11 (NI-siderophore) and 1.12 (terpene)	-
7	+	3443-3450	6	Located in region without BGCs	-
7	-	780130-780137	1	Between clusters 1.4 (terpene-hopene) and 1.5 (NRPS-holomycin)	-
6	-	1203755-1203762	1	In cluster 1.7 between genes *ctg1_1004* and *ctg1_10015*	Collismycin A (77%)
6	-	7222098-7222105	1	Between clusters 1.21 (terpene-isorenieratene) and 1.22 (T3PKS-naringenin)	-
5	+	2260-2267	6	Located in region without BGCs	-
2	+	177-184	6	Located in region without BGCs	-
2	+	780129-780136	1	Between clusters 1.4 (terpene-hopene) and 1.5 (NRPS-holomycin)	-
2	-	4136083-4136090	1	Between clusters 1.16 (T2PKS-granaticin) and 1.17 (NI-siderophore-desferrioxamin B)	-

*Based on AntiSMASH predictions

Table S3: motifs located in *Streptomyces* sp. CS057

Motif	Strand	Position	Scaffold	Placed in	Within a BGC*
18	+	3962476-3962483	1	Between clusters 1.22 (T1PKS/NRPS-like) and 1.23 (NRPS-skyllamycin D, E)	-
14	+/-	1530265-1530272	1	Between clusters 1.12 (terpene) and 1.13 (RiPP-like)	-
14	+/-	4182780-4182787	1	Between clusters 1.23 (NRPS-skyllamycin D, E) and 1.24 (ectoine)	-
14	+/-	5730986-5730993	1	Between clusters 1.25 (NI-siderophore-desferrioxamin B) and 1.26 (lanthipeptide clas iii/lanthipeptide class ii)	-
13	+	3786849-3786856	1	Between clusters 1.22 (T1PKS/NRPS-like) and 1.23 (NRPS-skyllamycin D, E)	-
11	-	2278817-2278824	1	Between clusters 1.16 (NI-siderophore) and 1.17 (terpene)	-
11	+	2750175-2750182	1	Between clusters 1.18 (triceptide) and 1.19 (lanthipeptide class iii-AmfS)	-
11	+	6632859-6632866	1	Between clusters 1.27 (lanthipeptide class ii/ NRPS) and 1.28 (ectoine)	-
11	+	7470016-7470023	1	Between clusters 1.29 (terpene) and 1.30 (NRPS)	**-**
9	-	8278882-8278889	1	In cluster 1.35. Gene *ctg1_6972*	Coelimycin P1 (8%)
8	-	897433-897440	1	In cluster 1.9 between genes *ctg1_707* and *ctg1_708*	SGR PTMs (100%)
8	-	1158659-1158666	1	Between clusters 1.11 (terpene-hopene) and 1.12 (terpene)	-
8	+	2253716-2253723	1	Between clusters 1.16 (NI-siderophore) and 1.17 (terpene)	-
8	-	7365147-7365154	1	Between clusters 1.29 (terpene) and 1.30 (NRPS)	**-**
8	-	7963726-7963733	1	In cluster 1.32 between genes *ctg1_6699* and *ctg1_6700*	Naringenin (100%)
7	+	2164-2171	3	Located in region without BGCs	**-**
5	+	981-988	3	Located in region without BGCs	**-**
2	+	4284-4291	3	Located in region without BGCs	**-**

*Based on AntiSMASH predictions

Table S3: Continuation

Motif	Strand	Position	Scaffold	Placed in	Within a BGC*
2	-	4674325-4674332	1	Between clusters 1.23 (NRPS-skyllamycin D, E) and 1.24 (ectoine)	-

*Based on AntiSMASH predictions

Table S4: motifs located in *Streptomyces* sp. CS065a.

Motif	Strand	Position	Scaffold	Placed in	Within a BGC*
16	+	253754-253761	2	Between clusters 1.4 (NRPS-like/NRPS) and 1.5 (RiPP-like)	-
14	+/-	1670222-1670229	1	Between clusters 1.12 (NI-siderophore) and 1.13 (terpene)	-
14	+/-	4868237-4868244	1	Between clusters 1.21 (LAP/thiopeptide) and 1.22 (NI-siderophore-desferrioxamin B)	-
11	-	6536948-6536955	1	Between clusters 1.26 (terpene) and 1.27 (T3PKS)	-
8	-	462480-462487	1	Between clusters 1.7 (NRPS) and 1.8 (terpene-hopene)	-
8	-	1640998-1641005	1	Between clusters 1.12 (NI-siderophore) and 1.13 (terpene)	-
8	-	2258349-2258356	1	Between clusters 1.14 (lanthipeptide class iii-AmfS) and 1.15 (lassopeptide-keywimysin)	-
8	+	6423237-6423244	1	Between clusters 1.25 (ectoine) and 1.26 (terpene)	-
7	+	3624-3631	3	Located in region without BGCs	-
7	-	5087510-5087517	1	Between clusters 1.23 (lanthipeptide class iii/lanthipeptide class ii) and 1.24 (T2PkS/oligosaccharide-chromomycin A3)	-
6	+	2856986-2856993	1	Between clusters 1.14 (lanthipeptide class iii-AmfS) and 1.15 (lassopeptide-keywimysin)	-
5	-	4807-4814	3	Located in region without BGCs	-

2	+	1504-1511	3	Located in region without BGCs	-
2	+	3214013-3214020	1	Between clusters 1.16 (nucleoside) and 1.17 (betalactone)	-

*Based on AntiSMASH predictions

Table S5: motifs located in *Streptomyces* sp. CS131.

Motif	Strand	Position	Scaffold	Placed in	Within a BGC*
18	+	2739764-2739771	1	Between clusters 1.12 (NI-siderophore-desferrioxamin B) and 1.13 (thiopeptide/LAP)	-
16	-	3145100-3145107	1	Between clusters 1.14 (NRPS-like) and 1.15 (NRPS)	-
15	+	4537763-4537770	1	Between clusters 1.19 (lanthipeptide class iv) and 1.20 (NRPS-like/T1PKS)	-
14	+/-	34989-34996	1	In cluster 1.1 between genes *ctg1_36* and *ctg1_37*	C-1027 (8%)
11	-	861714-861721	1	Between clusters 1.7 (terpene-isorenieratene) and 1.8 (NRPS)	-
9	-	4110550-4110557	1	Between clusters 1.17 (lanthipeptide class ii) and 1.18(NRPS/arylpolyene)	-
8	-	5886475-5886482	1	Between clusters 1.23 (terpene) and 1.24 (NI-siderophore)	-
8	-	6368090-6368097	1	Between clusters 1.26 (lanthipeptide class i) and 1.27 (RiPP-like)	-
7	+	1707-1714	5	Located in region without BGCs	-
7	+	3860513-3860520	1	Between clusters 1.16 (lassopeptide-stlassin) and 1.17 (lanthipeptide class ii)	-
6	-	4110551-4110558	1	Between clusters 1.17 (lanthipeptide class ii) and 1.18 (NRPS/arylpolyene)	-
5	+	524-531	5	Located in region without BGCs	-
4	+	840481-840488	1	Between clusters 1.7 (terpene-isorenieratene) and 1.8 (NRPS)	-
2	+	3827-3834	5	Located in region without BGCs	-

2	+	3664251-3664258	1	Between clusters 1.16 (lassopeptide-stlassin) and 1.17 (lanthipeptide)	-

*Based on AntiSMASH predictions

Table S6: motifs located in *Streptomyces* sp. CS227.

Motif	Strand	Position	Scaffold	Placed in	Within a BGC*
16	-	2496275-2496282	1	Between clusters 1.11 (terpene) and 1.12 (LAP/thiopeptide)	-
16	+	3888292-3888299	1	Between clusters 1.15 (NRPS) and 1.16 (NRPS-surugamide A/surugamide D)	-
14	+/-	1640279-1640286	1	Between clusters 1.9 (NI-siderophore) and 1.10 (terpene-geosmin)	-
13	-	2715534-2715541	1	In cluster 1.13 between genes *ctg1_orf04382* and *ctg1_orf04384*	Not provided (RiPP-like)
7	+	3788-3795	6	Located in region without BGCs	-
5	+	2605-2612	6	Located in region without BGCs	-
2	+	522-529	6	Located in region without BGCs	-
2	-	3626762-3626769	1	Between clusters 1.15 and 1.16	-

*Based on AntiSMASH predictions

LC-MS dereplication

CS014:

Collismycin A-B, $C_{13}H_{13}N_3O_2S$:

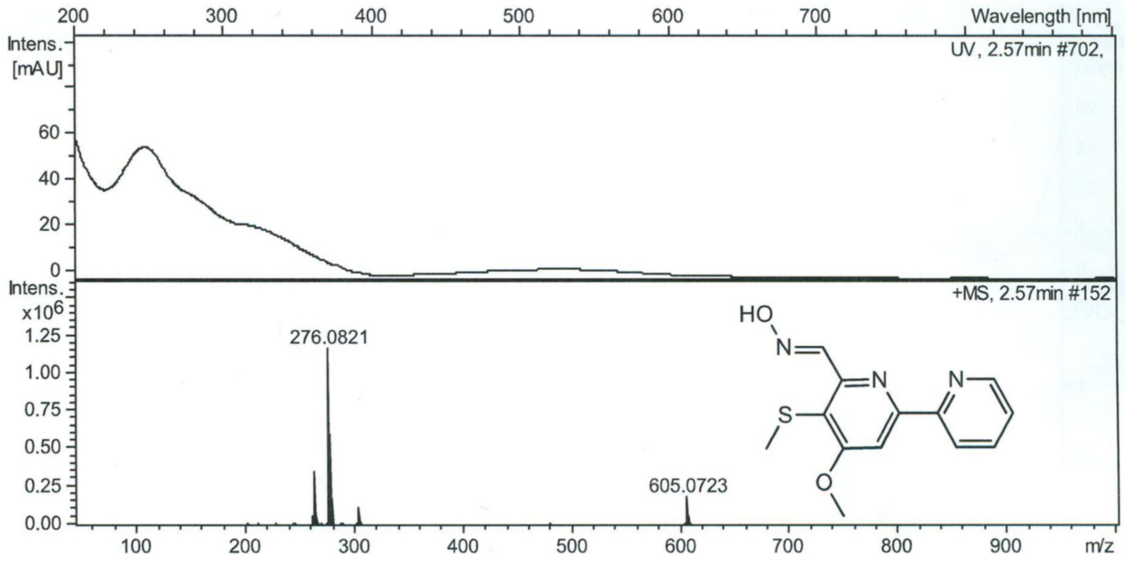

Collismycin C, $C_{13}H_{14}N_2O_2S$:

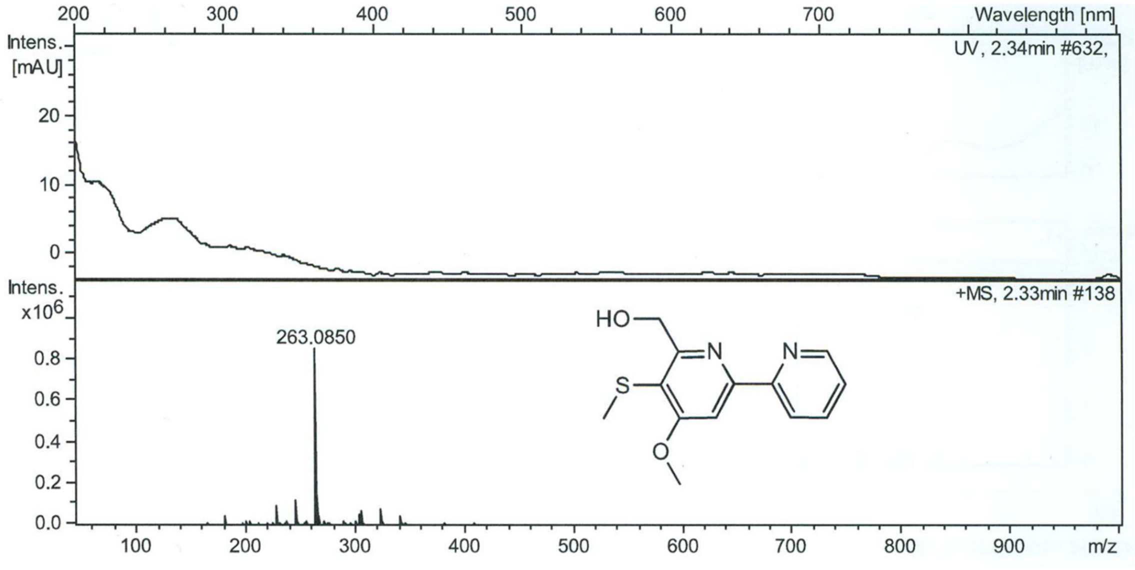

Collismycin D, $C_{13}H_{11}N_3OS$:

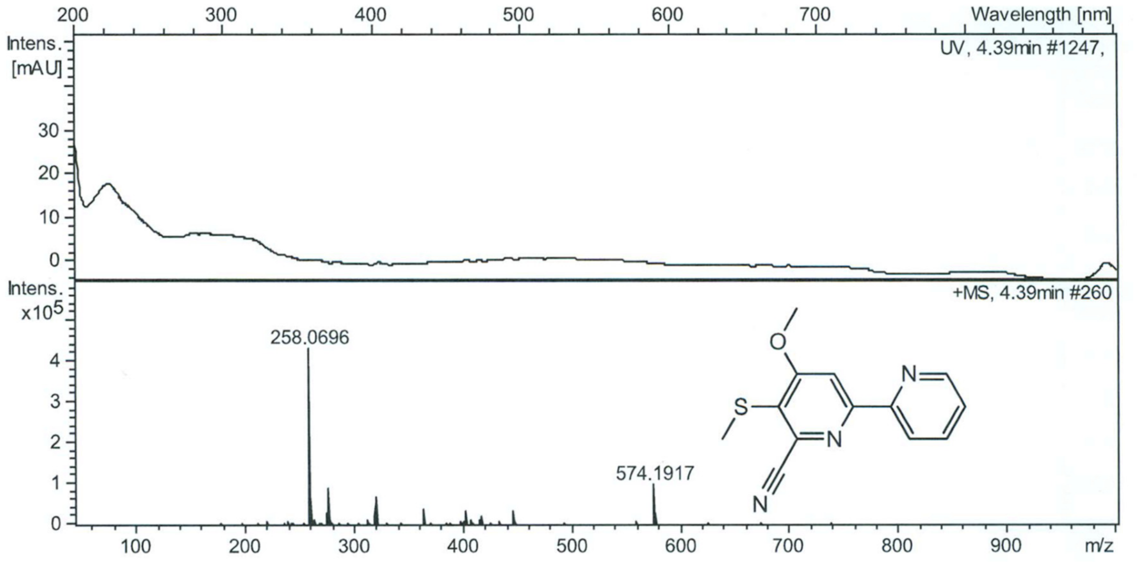

Granaticin A, $C_{22}H_{20}O_{10}$:

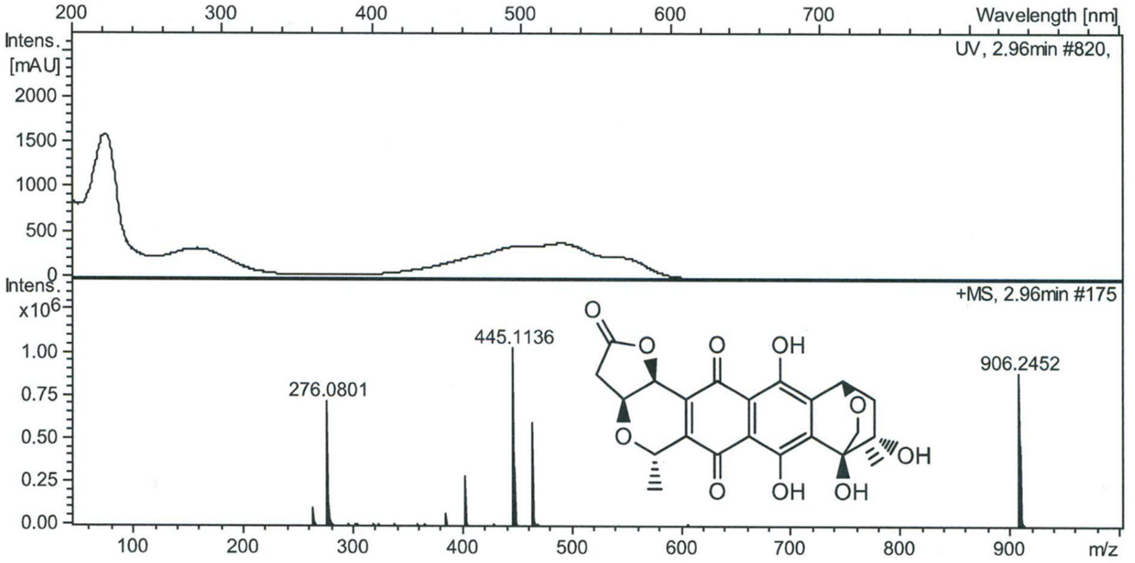

Granaticin C, $C_{28}H_{30}O_{12}$:

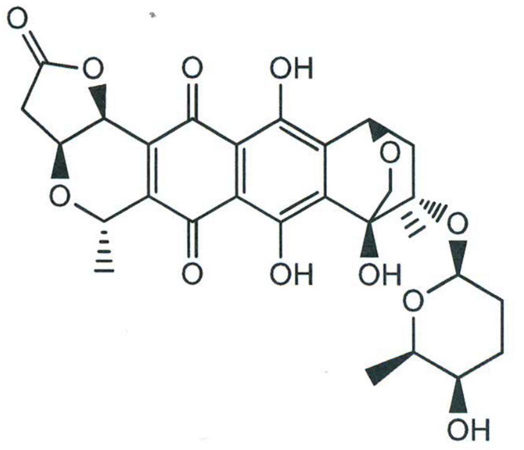

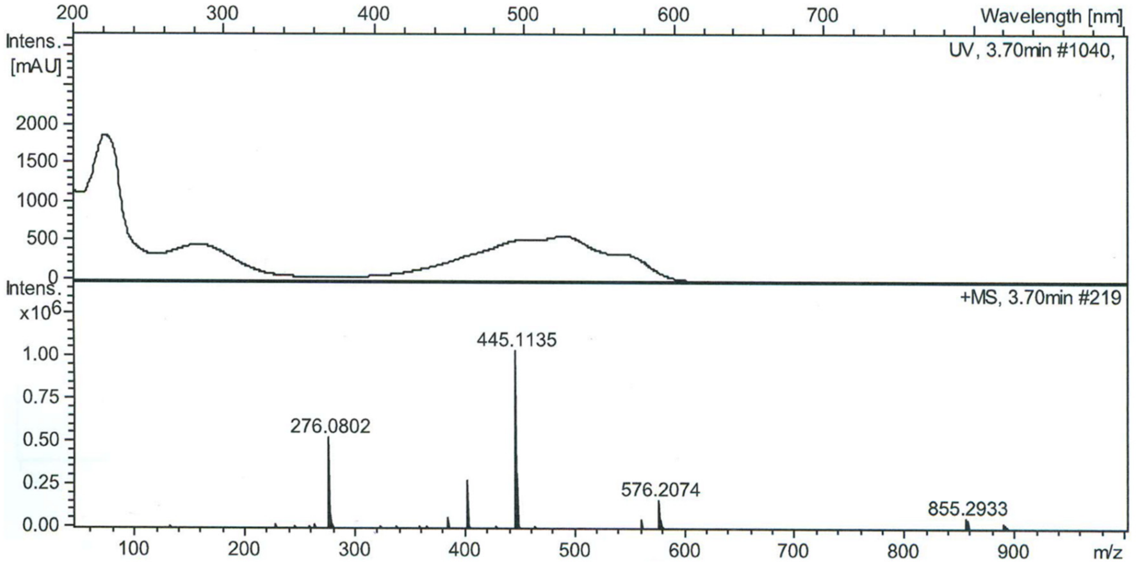

Coproporphyrin, $C_{36}H_{38}N_4O_8$

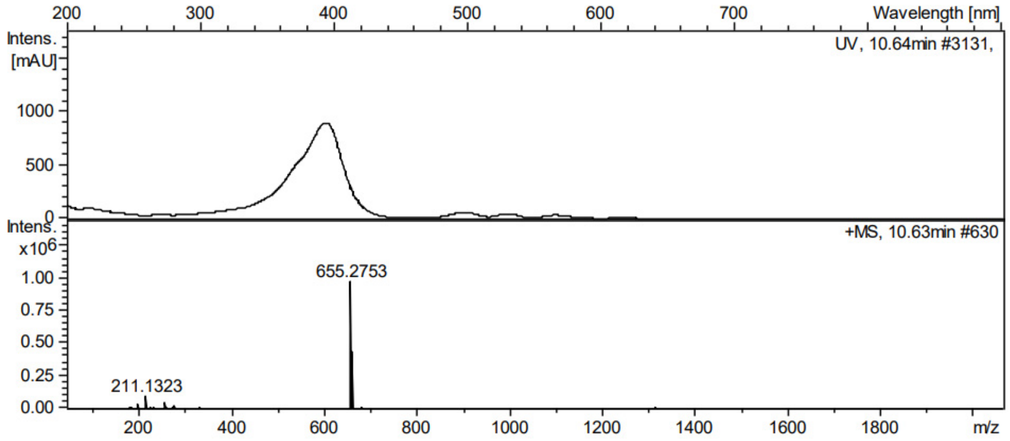

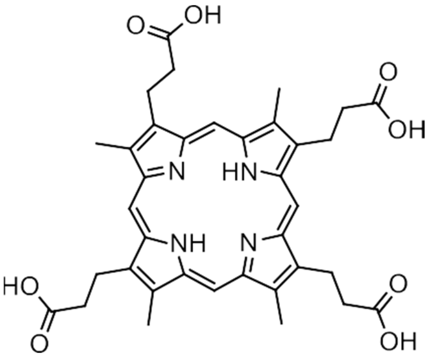

Cyclo (Tyr-Pro): $C_{14}H_{16}O_3$

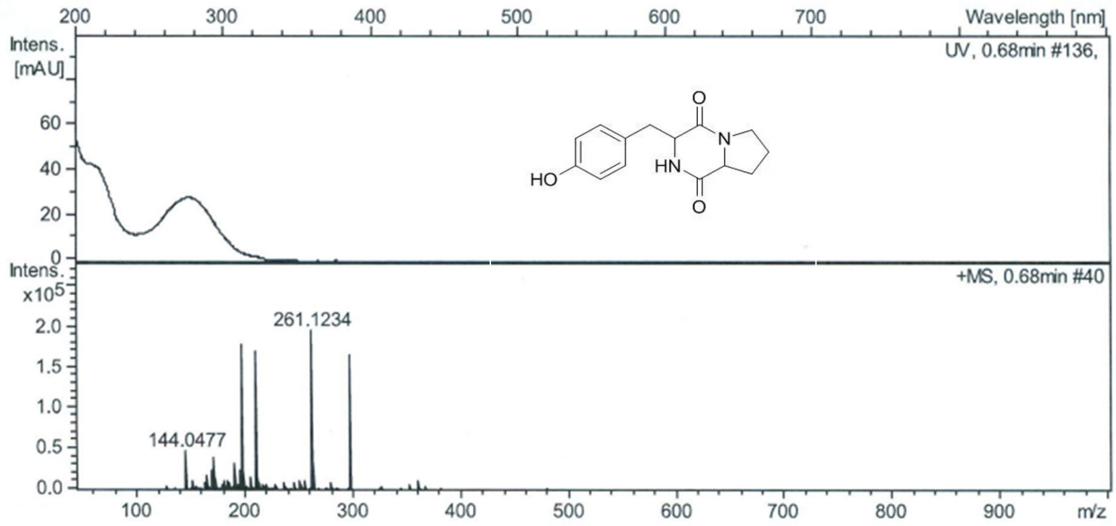

Aloesaponarin II: $C_{15}H_{10}O_4$

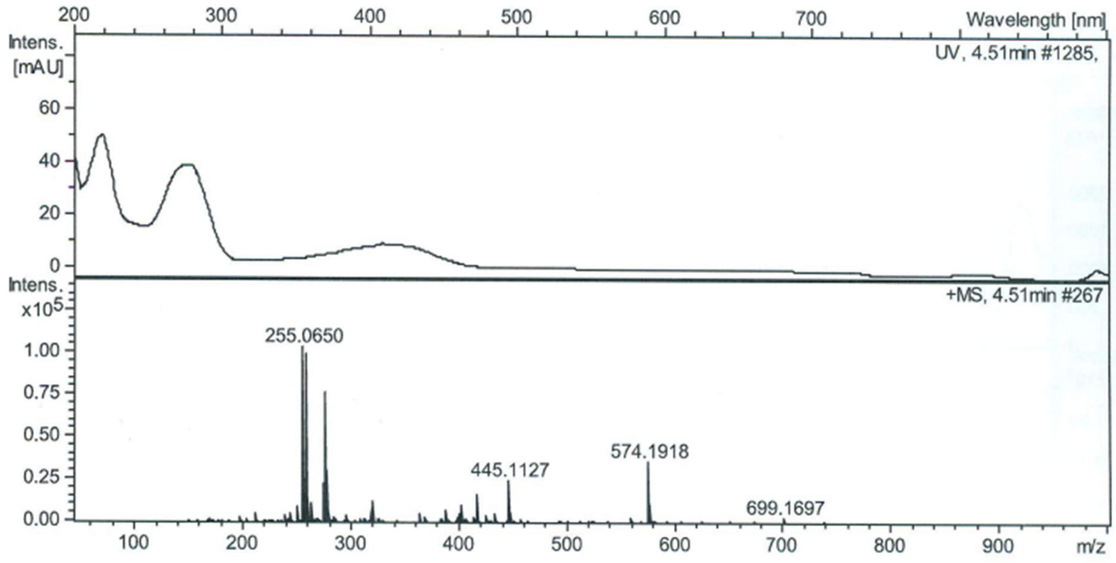

CS057:

Warkmycins: $C_{50}H_{67}NO_{23}$

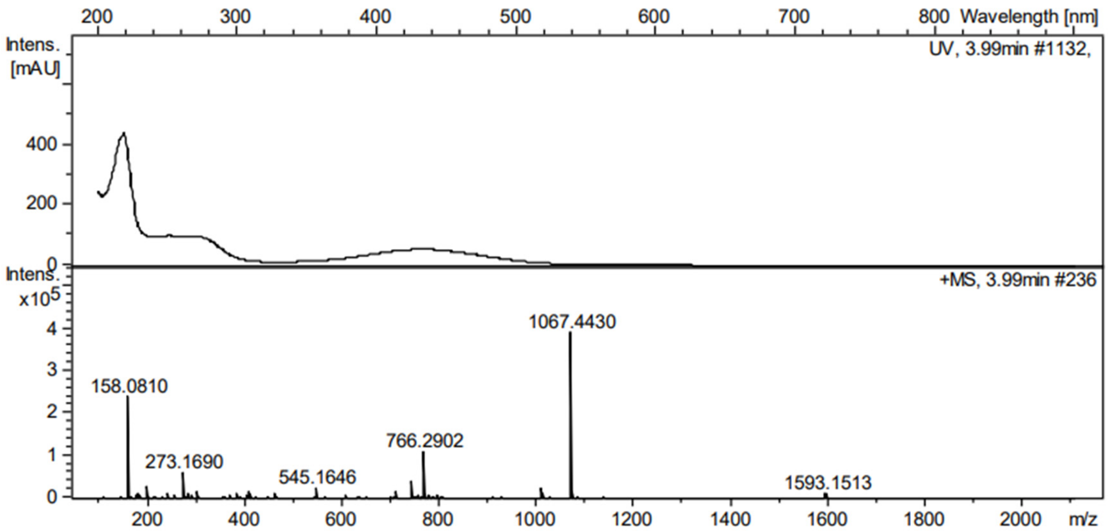

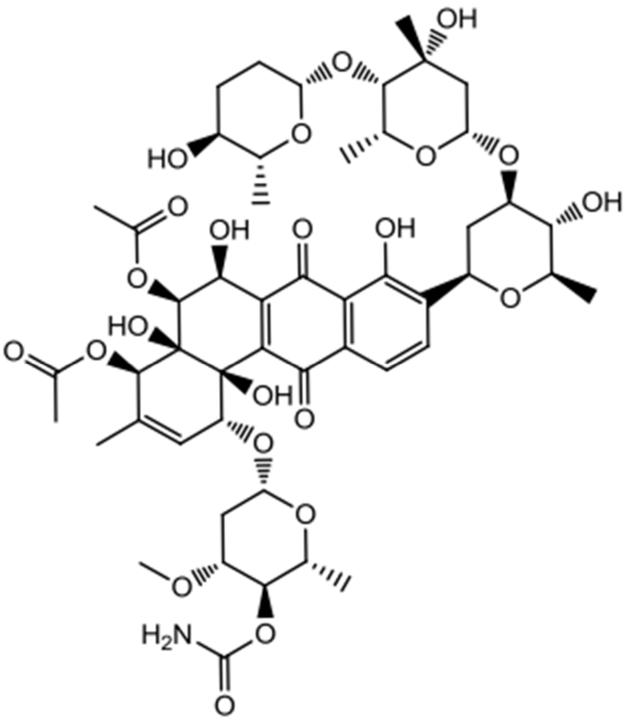

2-aminobenzoic acid: $C_7H_7NO_2$

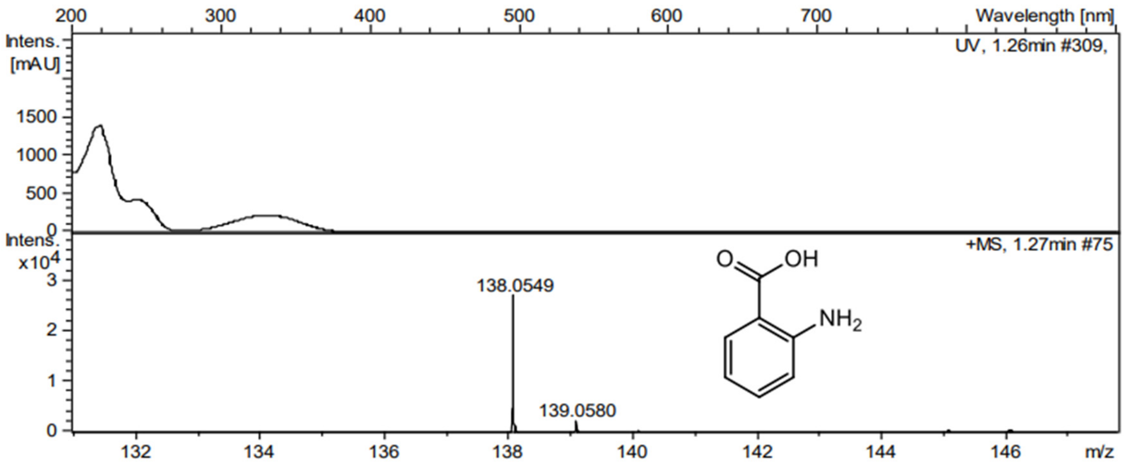

CS065:

Alteramide A: $C_{29}H_{38}N_2O_6$:

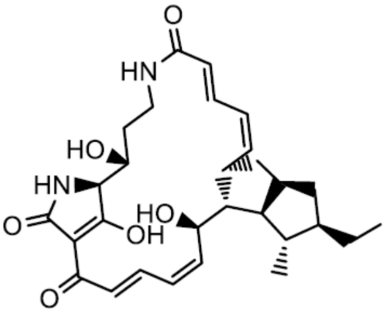

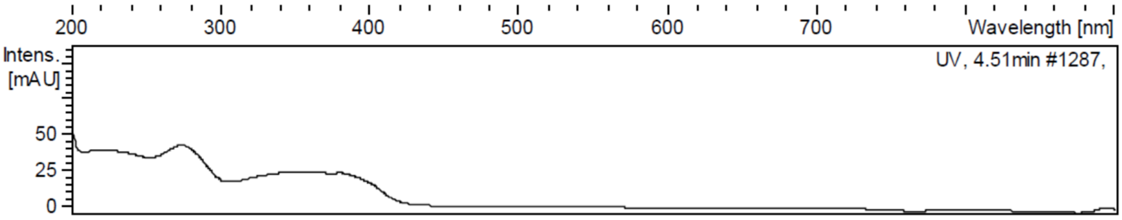

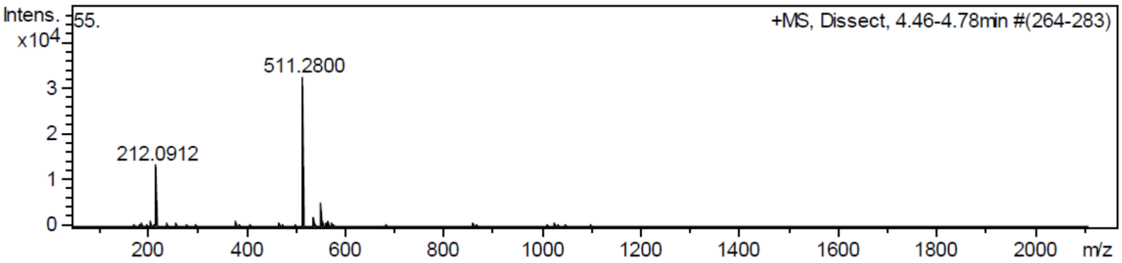

Chromomycin A3: $C_{57}H_{82}O_{26}$:

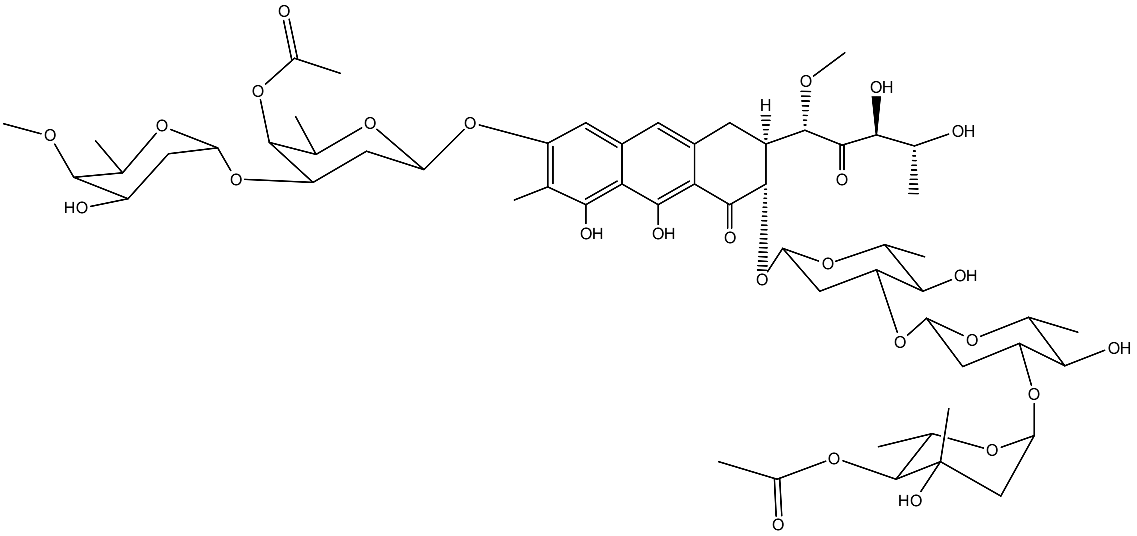

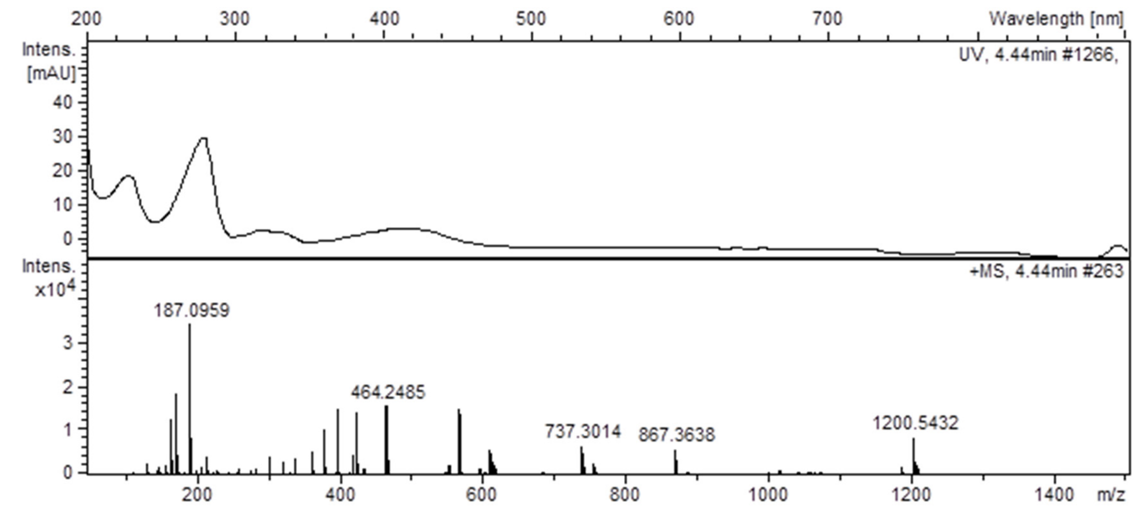

CS131:

Actinomycin: $C_{62}H_{86}N_{12}O_{16}$

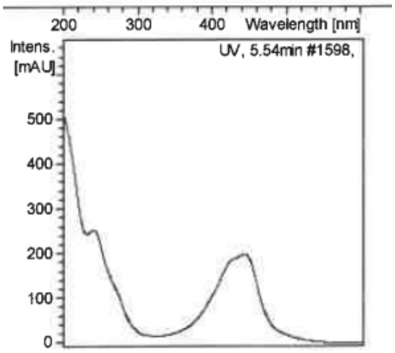

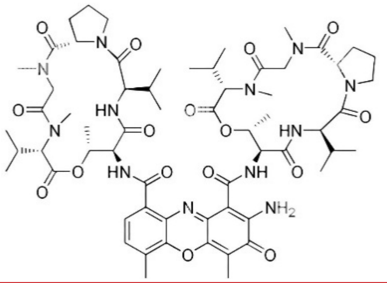

CS227:

Antimycin: $C_{27}H_{38}N_2O_9$

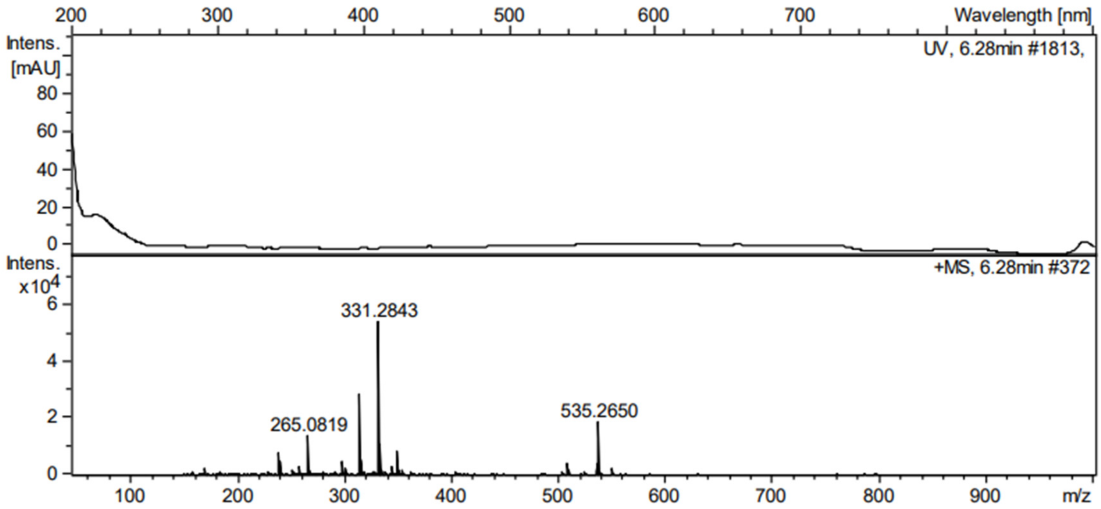

Candicidin: $C_{59}H_{84}N_2O_{18}$

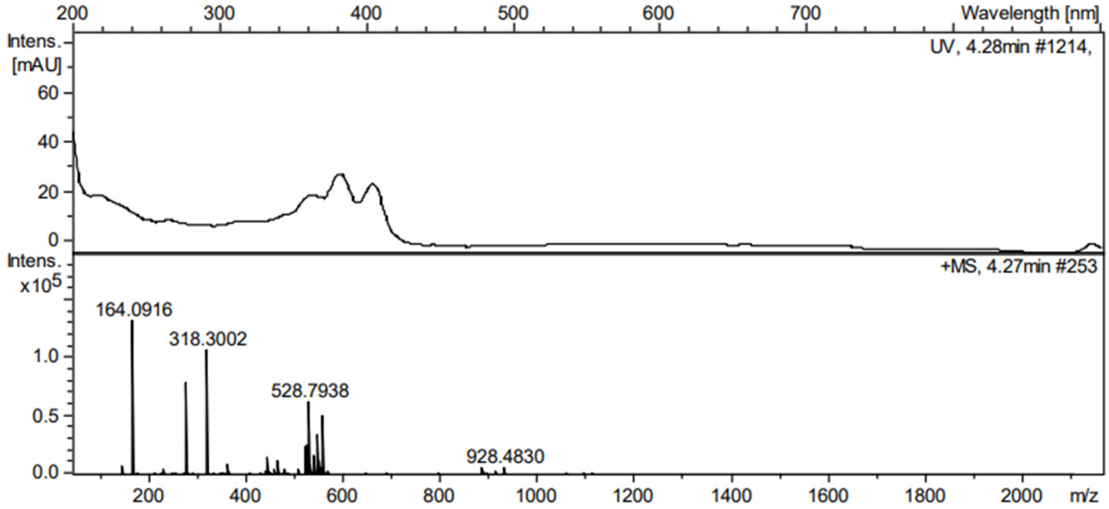

Alteramide: $C_{29}H_{38}N_2O_6$:

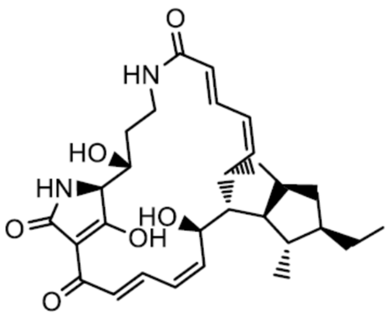

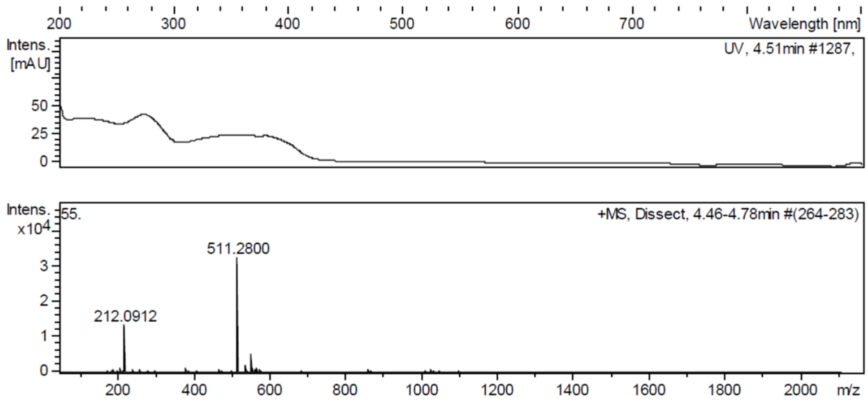

Dihydromaltophilin: $C_{29}H_{40}N_2O_6$

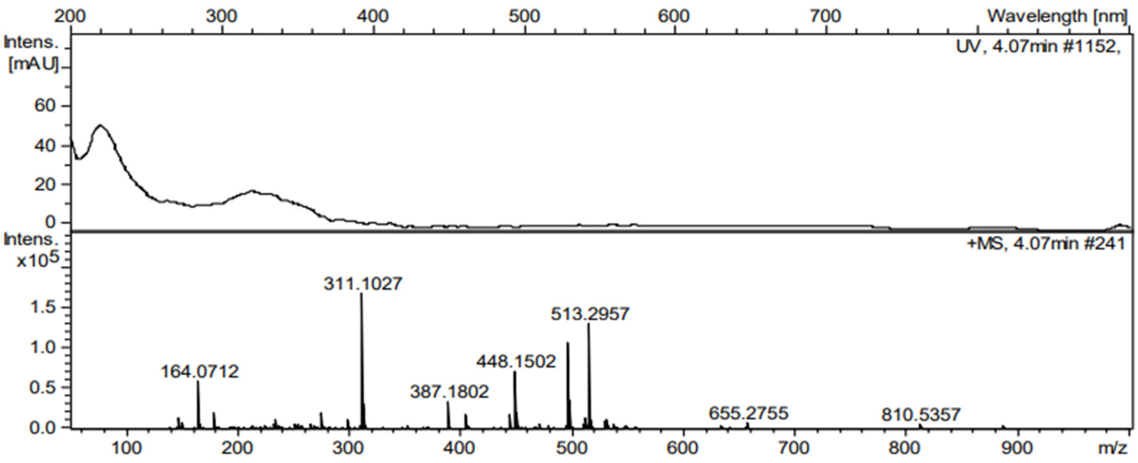

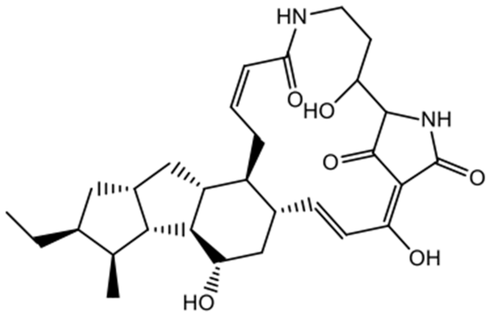

Supplement: Supplementary file 1 [file microorganisms-12-02317-s001.zip › microorganisms-3237691-supplementary.pdf]
